# Trace Biogenic Amine Analysis with Pre-Column Derivatization and with Fluorescent and Chemiluminescent Detection in HPLC

**DOI:** 10.6028/jres.093.137

**Published:** 1988-06-01

**Authors:** T. Kawasaki, O. Wong, C. Wang, T. Kuwana

**Affiliations:** Center for Bioanalytical Research, University of Kansas, Lawrence, KS 66046

## Background

Research at the Center for Bioanalytical Research (CBAR) is aimed toward the development of highly sensitive and selective methods for the analysis of biological substances. The late Professor Takeru Higuchi, in creating the Center in 1983, initially focussed on target analytes such as amines, amino acids, peptides and polypeptides. Although methods such as RIA provide sensitive and selective analysis, a decision was made to develop fluorescence, chemiluminescence and electrochemistry for detection and liquid chromatography for separation; i.e. selectivity. Since most amino acids and peptides are optically and electrochemically “silent,” it is necessary to derivatize these substances so that they can be detected. The objective of the present work was to develop a derivatization scheme with LC separation and with fluorescence (FL) and chemiluminescence (CL) detection of catecholamines and other biogenic amines in biological samples.

It is well known that electrochemical (EC) methods offer high sensitivity detection for LC analysis of catecholamines in biological samples [1–5]. Luminescence detection methods, such as FL and CL, are also capable of providing high sensitivity detection [6–9], particularly when the weakly fluorescing amines are fluorescent labelled [8–14] by derivatization. Fluorogenic reagents such as ortho-phthalaldehyde (OPA) and fluorescamine (FCA), which are specific for primary amines [15, 16], have been used to enhance the sensitivity of catecholamines for LC analyses. The OPA method has been significantly improved by CBAR with the development of a new fluorogenic reagent, 2,3-naphthalenecarboxaIdehyde (I, NDA), which in the presence of cyanide ion, reacts with primary amines to give the intensely fluorescent product, 1-cyano-2-substituted-benz[f]isoindoles (II, CBI) [18].

**Figure:**
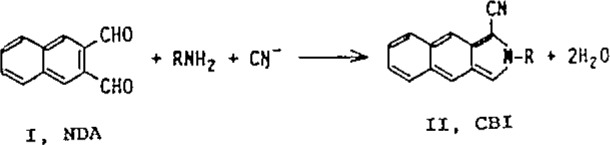


The stability and FL quantum yields of the CBI-amine derivatives were superior to those from the OPA-thiol reaction [18]. The reaction of NDA with dopamine (DA), norepinephrine (NE), and the trace amines, octopamine (OA) and tyramine (TA), as a pre-column fluorogenic LC derivatization method, for the simultaneous determination of DA and NE in urine has been studied.

## Experimental

Instrumentation: Optical absorbance, fluorescence and chemiluminescence measurements were made with a Shimadzu Model UV-260 spectrophotometer, a Farrand System 3 scanning spectrofluorometer, and an ATTO Model AC220 luminometer, respectively. The isocratic LC system consisted of a LKB Model 2150 dual-piston pump equipped with a Rheodyne 7125 sample injector with a 5 μL sample loop. For some of the LCCL studies, an ISCO Model 314 syringe pump was used.

Chromatography and Derivatization Procedures: CBI derivatives of catecholamine standards were separated using a TSK ODS-120T column (4.6 × 150 mm, 5 *μ*m). The mobile phase was 50% acetonitrile in 10 mM potassium phosphate (pH 2.5, adjusted with phosphoric acid). The flow rate was 1 mL/min. For DA and NE analysis in urine samples, the mobile phase was isocratic elution with 38% acetonitrile and 6% THF in 10 mM phosphate buffer at pH 2.5. Derivatizations were conducted in borate buffer with dihydroxybenzylamine (DHBA) as an internal standard. Final concentrations were [NDA] = 0.2 mM, [CN] = 0.2 mM and [DHBA] = 5 μM. For the urine samples, alumina was used for sample clean-up. Desorption of catecholamines from alumina was accomplished by washing (30 seconds) the alumina with two successive portions (200 *μ*L) of 0.1 M phosphoric acid.

## Results and Discussion

**Figure 1:**
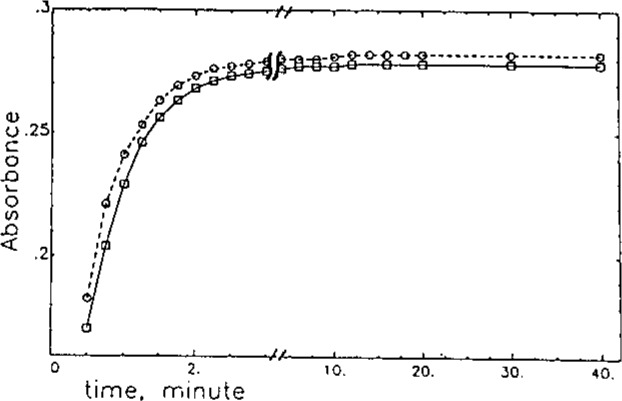
Absorbance-time profile for the reaction of NDA-CN with DA and NE.

**Figure 2:**
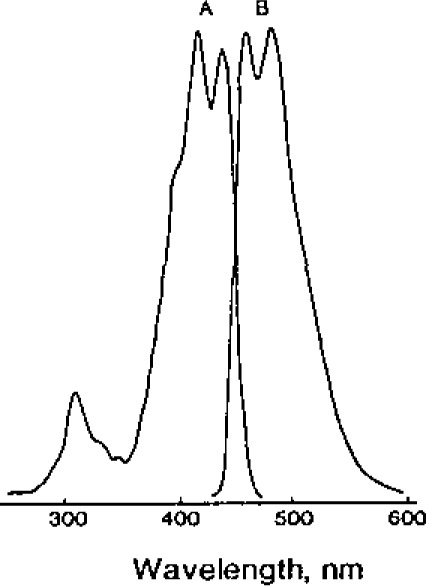
Typical optical absorbance (A) and fluorescence emission (B) spectra of a CBI-amine derivative.

Figure 1 shows the absorbance-time profile for the reaction of NDA-CN with DA and NE in borate buffer (pH 9) at room temperature. Under these conditions, the reaction is completed in less than 5 minutes with the CBI-catecholamines exhibiting excellent chemical stability. The rapidity and completeness of the NDA-CN reaction under mild aqueous conditions are an important feature compared to that of the fluorescamine method [16], which requires the fluorescamine reagent to be solubilized in an anhydrous organic solvent to prevent deleterious hydrolysis from occurring. Also, compared to the usual OPA-thiol method [15], the CBI product has enhanced chemical stability. Figure 2 shows typical optical (A) absorbance and (B) fluorescence emission spectra of a CBI-amine derivative. The optical absorbance in the visible region of the spectrum allows the use of a low-powered He-Cd laser for excitation, which is currently under development [21].

**Figure 3:**
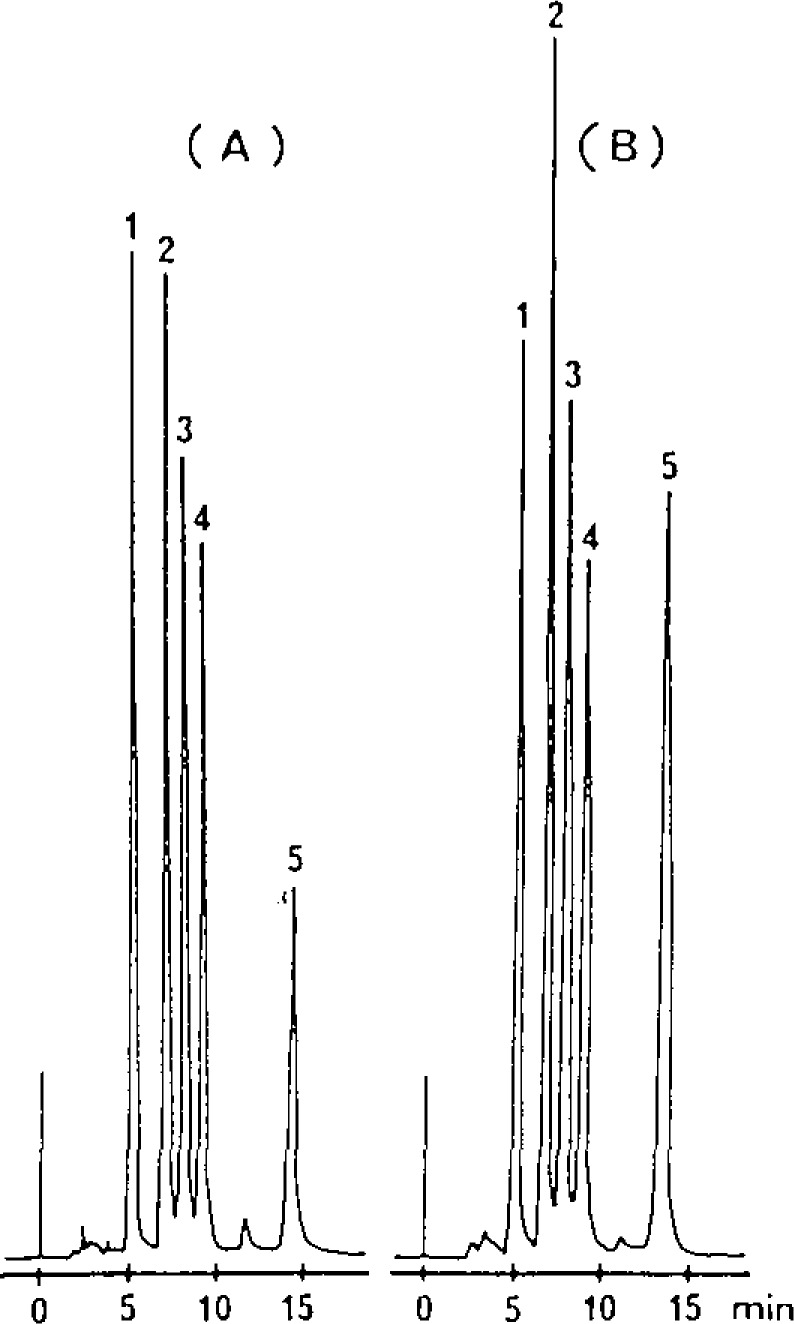
Isocratic reverse-phase LC separation of five CBI derivative (A). Same separation after addition of SDS (B).

Figure 3A shows the separation of a mixture of five CBI derivatives by reverse phase LC under isocratic conditions. The five derivatives were separated to baseline resolution within 15 minutes. Addition of 10 mM SDS in the mobile phase enhanced the FL intensities of the trace amines, CBI-OA and CBI-TA but had little effect on the other three (fig. 3B). Using DHBA as the internal standard, good linear response (linear regression correlation coefficient >0.995) was obtained in the range of 0.5 to 10 pmol of amine injected on-column. Detection limits for these amines are in the range of 20–60 femtomoles (S/N=2) with the commercial 10 μL FL detector.

Chromatograms for the determination of DA and NE in 1 μL urine samples containing DHBA as the internal standard with the NDA-CN procedure indicate that these substances can be quantified at less than 0.5 μM concentrations.

## Chemiluminescence

Chemiluminescent detection of the CBI-catecholamines used bis(2,4-dinitrophenyl)oxalate (DNPO) as the oxalate. The hydrogen peroxide and DNPO in acetonitrile solvent are post-column mixed the LC eluant and the CL emission detected with the ATTO cell of 40 or 60 μL volume. The sensitivity of the CL system is demonstrated by the baseline separation of a mixture containing 1 femtomole each of DA, NE and DHBA. Calibration curves with DHBA as the standard exhibit excellent linearity over two orders of magnitude with a detection limit of 0.01 pg.
